# A GTP-driven central carbon metabolism in the cellulolytic bacterium *Ruminiclostridium cellulolyticum*

**DOI:** 10.1038/s42003-025-07971-7

**Published:** 2025-03-30

**Authors:** Nian Liu, Nicolas Vita, Marion Holmière, Séverine Gagnot, Gaël Brasseur, Pascale de Philip, Sandrine Pagès, Stéphanie Perret, Henri-Pierre Fierobe

**Affiliations:** 1 Aix Marseille Univ, CNRS LCB, Marseille, France; 2 Present Address: Baylor College of Medicine, Houston, TX USA

**Keywords:** Cellular microbiology, Kinases

## Abstract

In *Ruminiclostridium cellulolyticum*, the hexokinase and galactokinase were formerly shown to strongly prefer GTP over ATP, whereas the phosphofructokinase is PPi-dependent, suggesting an unconventional central carbon metabolism in this anaerobic bacterium. Herein, the characterization of all other kinases of this pivotal pathway led to the identification of their preferred NTP/NDP. The kinases involved in the first reactions, primarily functioning as NTP-consuming enzymes, appear to be GTP-dependent. In contrast, the enzymes catalyzing the downstream steps that mainly generate NTP, show no marked preference. Consequently, its central carbon metabolism appears essentially driven by GTP, whose cellular content nears that of ATP. Interestingly, in vivo reciprocal exchange of the GTP-dependent hexokinase in *R. cellulolyticum* by the ATP-dependent glucokinase from *Escherichia coli* and vice versa generates modified strains that still catabolize glucose and glucose disaccharides. Altogether our data suggest an unexpected diversity and flexibility in the functioning of this central pathway in bacteria.

## Abstract (web summary)

Characterizing all kinases involved in central carbon metabolism of the cellulosome-producing bacterium Ruminiclostridium cellulolyticum shows that this pathway is unconventional and exhibits NTP flexibility.

## Introduction

Central carbon metabolism (CCM) designates the conversion of sugars into metabolic precursors and energy in heterotrophs. It encompasses the Embden-Meyerhof-Parnas (EMP) pathway of glycolysis, the pentose phosphate pathway, and includes the tricarboxylic acid (TCA) cycle in aerobic organisms as the final step. In anaerobiosis, the pyruvate produced through the glycolytic pathway is converted into reduced products like acids and/or alcohols, together with H_2_ and CO_2_. CCM is assumed to be ATP-driven and extensive investigations in model prokaryotes (*Escherichia coli*, *Bacillus subtilis*, *Brucellae*, etc.)^1–3^ and eukaryotes (yeast, etc.)^4^ revealed a common way to operate, relying on metabolic enzymes displaying similar properties^5–7^. These data were exploited to construct metabolic models^8,9^.

Nevertheless, few studies report the involvement of non-canonical enzymes in the CCM like the 3-phosphoglycerate kinase (7th step of glycolysis) from the human parasite *Entamoeba histolytica*, which catalyzes the reversible phosphoryl transfer from 1,3-biphosphoglycerate to mainly GDP (and not ADP) to synthesize GTP and 3-phosphoglycerate^10^. Likewise, the glucokinase (GLK) (1st step of glycolysis) of the cellulolytic thermophile *Acetivibrio thermocellus*, favors GTP over ATP as the phosphate donor to convert glucose into glucose-6P, and one of its phosphofructokinases (PFK) (3rd step of glycolysis) uses pyrophosphate (PPi) instead of ATP to generate fructose-1,6-biphosphate from fructose-6P^11,12^.

**Fig. 1:**
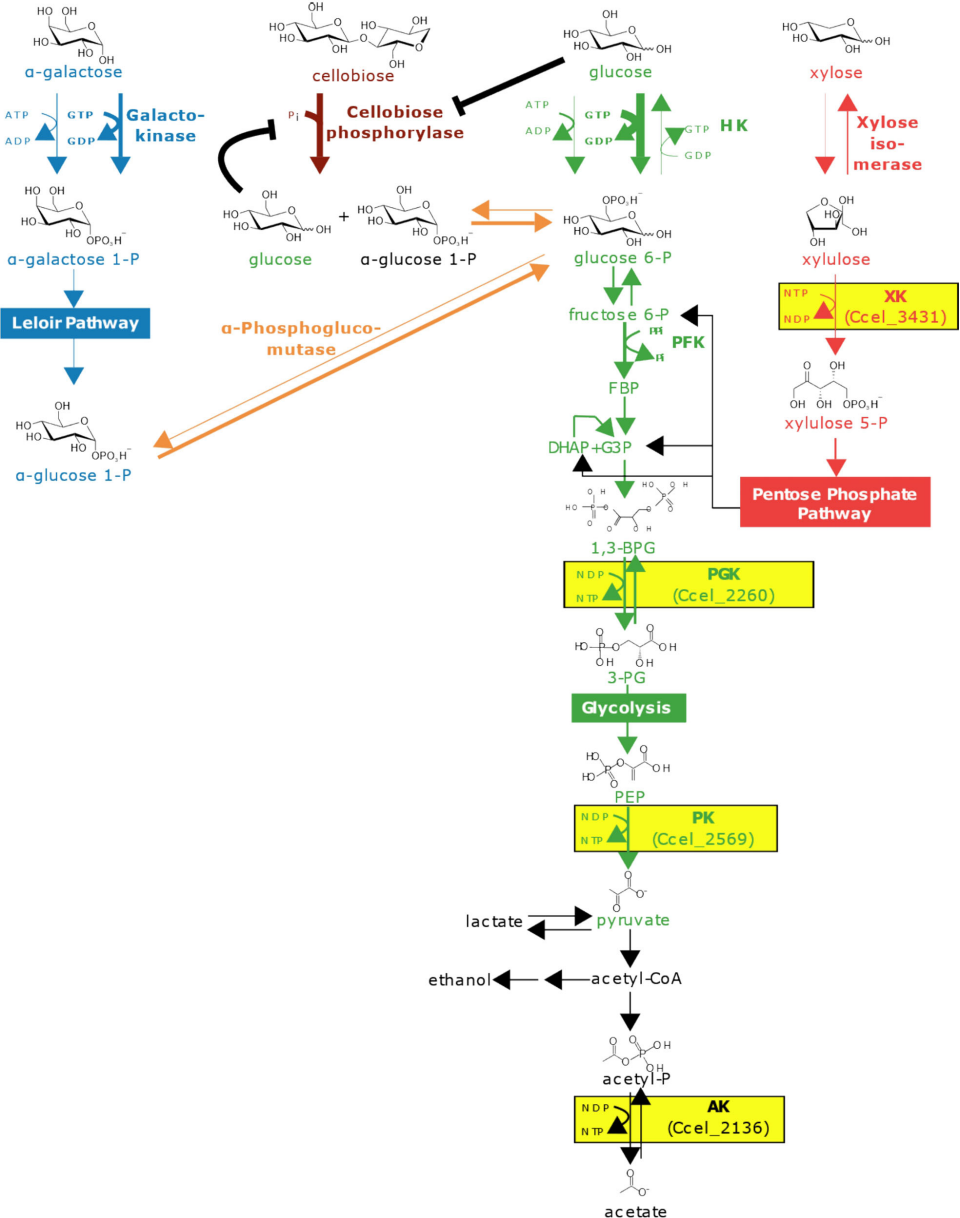
Schematic representation of the central carbon metabolism (CCM) in *R. cellulolyticum* and targeted enzymes. Formerly characterized enzymes involved in CCM^13,15^ are in bold. The kinases targeted in the present study are shaded in yellow and the locus number of their corresponding gene is indicated. XK xylulokinase, HK hexokinase, PFK phosphofructokinase, PGK phosphoglycerate kinase, PK pyruvate kinase, AK acetate kinase, FBP fructose-1,6-biphosphate, DHAP dihydroxyacetone phosphate, G3P glyceraldehyde-3-phosphate, 1,3-BPG 1,3-biphosphoglycerate, 3-PG 3-phosphoglycerate, PEP phosphoenol pyruvate.

As observed for *A. thermocellus*, the PFK activity detected in crude extracts of the cellulolytic and mesophilic anaerobe *Ruminiclostridium cellulolyticum*, is also PPi-dependent^13^ (Fig. 1). Furthermore, previous characterization of *R. cellulolyticum* galactokinase which converts α-galactose into α-galactose-1P (1st step of Leloir pathway), and of the enzyme which phosphorylates the 6th carbon of glucose (1st step of glycolysis) but also that of mannose, which classifies it as a hexokinase (HK), showed that they both display a marked preference (up to 100-fold) for GTP over ATP^14,15^ (Fig. 1). For both enzymes, this preference is principally due to much lower *K*_M_ values for GTP (<1 mM), compared with that for ATP (>10 mM). Additionally, it was also shown in vitro that the depletion of the GTP pool induces a reversion of the first step of glycolysis performed by the hexokinase^15^.

Altogether, the above-mentioned data suggest an unconventional CCM in cellulolytic anaerobic bacteria such as *R. cellulolyticum*, which may be GTP- and not ATP-driven, in contrast to most other known organisms. Firstly, to assess this hypothesis, all uncharacterized kinases involved in the CCM in *R. cellulolyticum* (Fig. 1) were overproduced, purified, and studied to determine their preferred NTP (or NDP). Secondly, the flexibility of this critical pathway concerning its NTP/NDP specificity was also challenged in vivo in both *R. cellulolyticum* and *Escherichia coli*.

## Results

### Identification, overproduction, and purification of the targeted kinases

To investigate the functioning of CCM in *R. cellulolyticum* in terms of purine nucleotide requirements, we have targeted the uncharacterized xylulokinase (XK), the phosphoglycerate kinase (PGK), the pyruvate kinase (PK) and the acetate kinase (AK) involved in this pathway (Fig. 1), the hexokinase (HK), galactokinase and phosphofructokinase (PFK) having been formerly described to be either GTP- or PPi-dependent^13,15^. Analysis of the genome indicates that all the selected enzymes are encoded by unique genes (Fig. S1). The PK-encoding gene (CCEL_RS12995/Ccel_2569) probably constitutes a monocistronic unit. The other selected genes are likely to form operonic units alongside other genes involved in CCM. Thus the genes coding for XK (CCEL_RS17225/Ccel_3431), a Repressor Orf Kinase family protein (CCEL_RS17220/Ccel_3430), and a xylose isomerase^15^ (CCEL_RS172215/Ccel_3429) possibly form a 3-gene operon. In the same way, the gene encoding PGK (CCEL_RS11420/Ccel_2260) is located 98 bases upstream of the gene coding the phosphoglycerate mutase (CCEL_RS11415/Ccel_2259) whereas the gene encoding AK (CCEL_RS10800/Ccel_2136) is located 47 bases downstream of the gene coding for the phosphotransacetylase (CCEL_RS10805/Ccel_2137) (Fig. S1).

The targeted kinases were grafted a C-terminal His tag and overproduced in the *E. coli* BL21 (DE3) strain. Purification was performed from the crude extracts using affinity chromatography on Ni-NTA resin followed by chromatography on an anion-exchanger column as previously described^15^. For PGK an additional gel filtration step was needed to achieve purification to homogeneity.

### Characterization of the selected kinases

Characterization of XK, which catalyzes the transfer of a phosphate group from NTP to the fifth carbon of xylulose^16^ (Fig. 1), revealed that similarly to HK and galactokinase, the enzyme exhibits a pronounced preference for GTP. At a saturating concentration of xylulose, the enzyme displays a 10-fold reduced *K*_m_ for GTP compared to that of ATP (Table 1, Fig. S2A), whereas the determined *k*_cat_ values remain constant regardless of the type of NTP used as co-substrate. At high NTP concentrations, this kinase displays slightly improved kinetic parameters for xylulose with GTP compared to ATP (Table 1, Fig. S2B).

**Table 1:** Kinetic parameters of selected kinases

Enzyme (short name) *direction*^a^	Substrate^b^ fixed co-substrate concentration^c^ (activator concentration)^d^			
Xylulokinase (XK)	GTP Xylulose, 2.5 mM	ATP Xylulose, 2.5 mM	Xylulose GTP, 10 mM	Xylulose ATP, 25 mM
*forward*	*k*_cat_ = 6540 ± 177	*k*_cat_ = 6653 ± 281	*k*_cat_ = 8788 ± 375	*k*_cat_ = 7489 ± 147
	*K*_m_ = 0.12 ± 0.02	*K*_m_ = 1.18 ± 0.18	*K*_m_ = 0.30 ± 0.07	*K*_m_ = 0.50 ± 0.05
Phosphoglycerate kinase (PGK)	GDP-^e^	ADP-		
*forward*	*k*_cat_ = 771 ± 20	*k*_cat_ = 733 ± 12		
	*K*_m_ = 0.135 ± 0.008	*K*_m_ = 0.08 ± 0.002		
	*n*_H_ = 1.63 ± 0.11	*n*_H_ = 4.03 ± 0.4		
	GTP 3-PG, 25 mM	ATP 3-PG, 4 mM	3-PG GTP, 25 mM	3-PG ATP, 10 mM
*reverse*	*k*_cat_ = 2831 ± 348	*k*_cat_ = 4256 ± 148	*k*_cat_ = 3273 ± 365	*k*_cat_ = 5767 ± 194
	*K*_m_ = 4.66 ± 1.24	*K*_m_ = 0.48 ± 0.07	*K*_m_ = 6.90 ± 1.44	*K*_m_ = 0.65 ± 0.08
Pyruvate kinase (PK)	GDP PEP, 10 mM (FBP, 0)	ADP PEP, 10 mM (FBP, 0)	PEP GDP, 5 mM (FBP, 0)	PEP ADP, 5 mM (FBP, 0)
*forward*	*k*_cat_ = 977 ± 48	*k*_cat_ = 9508 ± 328	*k*_cat_ = 3084 ± 312	*k*_cat_ = 9212 ± 50
	*K*_m_ = 0.90 ± 0.14	*K*_m_ = 1.87 ± 0.14	*K*_m_ = 23.0 ± 4.0	*K*_m_ = 1.56 ± 0.02
		*n*_H_ = 1.41 ± 0.09^f^		*n*_H_ = 1.32 ± 0.02
	GDP PEP, 10 mM (FBP, 0.5 mM)	ADP PEP, 10 mM (FBP, 0.5 mM)	PEP GDP, 5 mM (FBP, 0.5 mM)	PEP ADP, 5 mM (FBP, 0.5 mM)
*forward*	*k*_cat_ = 2341 ± 102	*k*_cat_ = 9213 ± 245	*k*_cat_ = 3794 ± 218	*k*_cat_ = 9814 ± 152
	*K*_m_ = 0.92 ± 0.09	*K*_m_ = 1.74 ± 0.11	*K*_m_ = 6.40 ± 0.81	*K*_m_ = 1.64 ± 0.07
	*n*_H_ = 1.40 ± 0.18	*n*_H_ = 1.39 ± 0.07	*n*_H_ = 1.24 ± 0.01	*n*_H_ = 1.26 ± 0.05
	GDP PEP, 10 mM (FBP, 3 mM)	ADP PEP, 10 mM (FBP, 3 mM)	PEP GDP, 5 mM (FBP, 3 mM)	PEP ADP, 5 mM (FBP, 3 mM)
*forward*	*k*_cat_ = 5747 ± 218	*k*_cat_ = 9032 ± 328	*k*_cat_ = 6545 ± 146	*k*_cat_ = 9585 ± 143
	*K*_m_ = 1.06 ± 0.1	*K*_m_ = 1.75 ± 0.14	*K*_m_ = 2.75 ± 0.16	*K*_m_ = 1.52 ± 0.06
	*n*_H_ = 1.34 ± 0.13	*n*_H_ = 1.47 ± 0.11	*n*_H_ = 1.28 ± 0.06	*n*_H_ = 1.40 ± 0.06
	GDP PEP, 10 mM (FBP, 10 mM)	ADP PEP, 10 mM (FBP, 10 mM)	PEP GDP, 5 mM (FBP, 10 mM)	PEP ADP, 5 mM (FBP, 10 mM)
*forward*	*k*_cat_ = 7479 ± 287	*k*_cat_ = 8846 ± 383	*k*_cat_ = 7747 ± 152	*k*_cat_ = 9335 ± 90
	*K*_m_ = 0.89 ± 0.07	*K*_m_ = 1.47 ± 0.14	*K*_m_ = 2.30 ± 0.12	*K*_m_ = 1.60 ± 0.04
	*n*_H_ = 1.42 ± 0.17	*n*_H_ = 1.57 ± 0.17	*n*_H_ = 1.50 ± 0.07	*n*_H_ = 1.34 ± 0.04
Acetate kinase (AK)	GDP Acetyl-P, 5 mM	ADP Acetyl-P, 5 mM	Acetyl-P GDP, 10 mM	Acetyl-P ADP, 10 mM
*forward*	*k*_cat_ = 35,365 ± 1,150	*k*_cat_ = 25,437 ± 1668	*k*_cat_ = 32,137 ± 823	*k*_cat_ = 25,112 ± 1755
	*K*_m_ = 1.47 ± 0.13	*K*_m_ = 1.13 ± 0.21	*K*_m_ = 1.28 ± 0.07	*K*_m_ = 1.49 ± 0.2
	GTP Acetate, 10 mM	ATP Acetate, 10 mM	Acetate GTP, 10 mM	Acetate ATP, 10 mM
*reverse*	NA^g^	*k*_cat_ = 16.68 ± 0.74		*k*_cat_ = 12.85 ± 0.85
		*K*_m_ = 1.47 ± 0.17	NA	*K*_m_ = 2.37 ± 0.38

^a^Designates the forward or reverse reaction related to the “glycolysis direction”.

^b^*k*_cat_ values are given in iu/µmol. *K*_m_ values are in mM. The data show the means and standard deviations of three replicates.

^c^The kinetics were performed at a fixed, saturating concentration of the co-substrate.

^d^For the pyruvate kinase, the kinetics were performed in presence of the allosteric activator FBP at 0, 0.5, 3, and 10 mM.

^e^For the phosphoglycerate kinase (forward direction), coupled assays using glyceraldehyde 3-P (at a fixed concentration) and GAPDH were performed. Therefore, the concentration of the real (co-) substrate of the enzyme (1,3-BPG) is not known.

^f^Designates the Hill coefficient, when cooperativity is observed. Values of *n*_H_ between 0.9 and 1.1 (no significant cooperativity) are not reported.

^g^No activity detected.

PGK catalyzes the reversible transfer of a phosphate group from 1,3-bisphosphoglycerate (1,3-BPG) to NDP producing 3-phosphoglycerate (3-PG) and NTP^17^. Since the substrate (in the “forward” glycolytic reaction) 1,3-BPG is not commercially available, we employed coupled assays with commercial glyceraldehyde 3-phosphate dehydrogenase (GAPDH) and glyceraldehyde-3-P (G3P) and monitored spectrophotometrically the NAD+ reduction, as formerly described^10^. From the measured kinetic parameters for NDP (Table 1 and Fig S2C), it appears that the enzyme has a modest preference for ADP versus GDP, reflected by a lower *K*_m_ value for ADP (80 µM) compared to that for GDP (135 µM), while the *k*_cat_ values are in a similar range. However, a strong cooperative effect was only observed with ADP. Remarkably, the catalysis of the reverse reaction involving the conversion of 3-PG into 1,3-BPG (assessed by monitoring the oxidation of NADH) displays significantly greater efficiency in the presence of ATP (Fig. S2D). At high NTP concentrations, the enzyme’s ‘catalytic efficiency’ (*k*_cat_/*K*_m_) toward 3-PG was 19-fold higher with ATP compared to GTP (Table 1). This preference for ATP over GTP was consistently observed at elevated concentrations of 3-PG, resulting in a 15-fold higher catalytic efficiency for ATP compared to GTP (Fig. S2E). Therefore, unlike the forward reaction, PGK exhibits a strong preference for ATP when catalyzing the reverse glycolytic reaction.

**Fig. 2:**
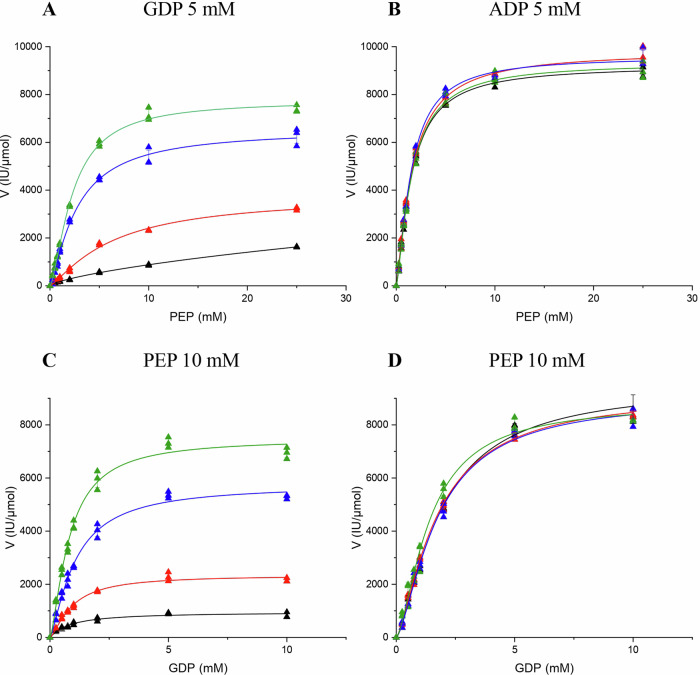
Nonlinear (Michaelis-Menten) regression analysis of the activities of the pyruvate kinase in the presence of 0 (back line), 0.5 mM (red line), 3 mM (blue line) and 10 mM (green line) fructose 1,6-biphosphate. **A** Analysis of the activity on PEP in the presence of 5 mM GDP. **B** Analysis of the activity on PEP in the presence of 5 mM ADP. **C** Analysis of the activity on GDP in the presence of 10 mM PEP. **D** Analysis of the activity on ADP in the presence of 10 mM PEP. The fixed co-substrate concentration is indicated on top of each graph. The data show the means of three independent experiments, and the bars indicate the standard deviations. Curves fitting was performed using the Origin 2019b software.

PK catalyzes the irreversible transfer of a phosphate group from phosphoenolpyruvate (PEP) to NDP, resulting in the formation of one molecule of pyruvate and one molecule of NTP^18^. Analysis of the sequence of the *R. cellulolyticum* PK indicates that it is a typical cluster-1 pyruvate kinase, suggesting its activity relies on K^+^ ion and is subject to allosteric regulation by fructose 1,6-bisphosphate (FBP)^6^. Surprisingly, its biochemical characterization with ADP revealed atypical features such as the absence of impact of FBP on the kinetic parameters for PEP which remain optimal (Table 1 and Fig. 2B). However, FBP was found to act as a potent allosteric activator only when GDP is the co-substrate. Indeed, increasing concentrations of FBP induce a drastic reduction of the *K*_m_ for PEP and the catalytic velocity improves until ultimately reaching, at the highest FBP concentration used (10 mM), values similar to those obtained in the presence of ADP (Fig. 2A, B, Table 1). Consistently, at saturating concentration of PEP, the presence of FBP induces an improvement of the catalytic velocity only when GDP is used (Table 1 and Fig. 2C), whereas the *K*_m_ value for the nucleotide remains unchanged, regardless of the concentration of FBP. Since the presence or absence of FBP had no impact on the kinetic parameters determined for ADP (Fig. 2D), *R. cellulolyticum* PK thus exhibits a clear preference for ADP, except when the glycolytic pathway is fully active. In this condition, FBP is released, which in turn drastically improves the catalytic parameters for PEP in the presence of GDP.

Finally, the characterization of AK catalyzing the transfer of a phosphate group from acetyl-P to NDP to generate acetate and NTP^19^, revealed that this enzyme does not display a marked preference for either GDP or ADP (Table 1, Fig. S2F). The enzyme exhibits similar *K*_m_ and *k*_cat_ values for GDP and ADP (Table 1) with kinetic parameters for acetyl-P within the same range at saturating concentrations of either GDP or ADP (Table 1 and Fig. S2G). However, it is worth noting that this enzyme exclusively catalyzes the reverse reaction when ATP serves as the co-substrate (Fig. S2H and I).

**Fig. 3:**
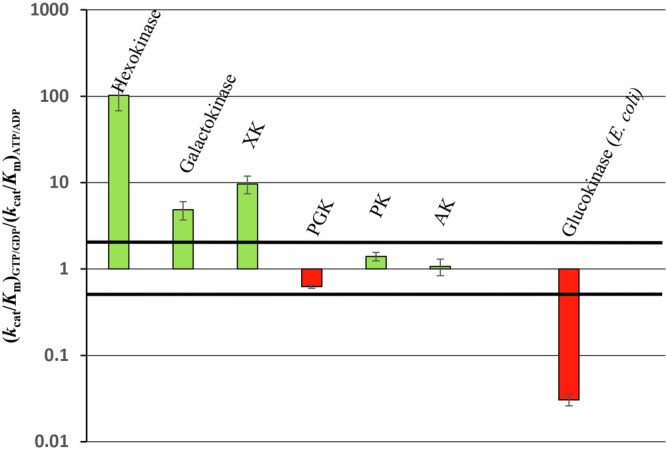
Graphical representation of the NTP/NDP selectivity of the various kinases. For each enzyme is reported the ratio (*k*_cat_/*K*_m_)_GTP/GDP_/(*k*_cat_/*K*_m_)_ATP/ADP_. The data were extracted from Table 1 “kinetic parameters of the selected kinases” and previous report^15^. For PK, the kinetic parameters determined in the presence of 10 mM FBP were used. The green and red bars correspond to the enzymes displaying a preference for GTP/GDP (ratio > 1) and ATP/ADP (ratio < 1), respectively. The black thick lines delimit an area in which no marked preference is observed, i.e., the ratio values are between 0.5 and 2.

Figure 3 depicts the ratios between the catalytic efficiencies (*k*_cat_/*K*_m_) for GTP or GDP and for ATP or ADP (considering only the forward reaction) calculated from the data related to the different kinases obtained in this work (Table 1) and previous studies^15^. The enzymes involved in sugar phosphorylation, especially HK, exhibit a pronounced preference for the co-substrate GTP, whereas the kinases catalyzing the downstream steps and generating NTP, show no significant preference for either GDP or ADP (Fig. 3, ratios ranging from 0.5 to 2). Taken together, these data indicate that the CCM in *R. cellulolyticum* is primarily GTP-driven since the GDP produced by the “upstream” kinases forces the “downstream” enzymes to use GDP as a cosubstrate and consequently to produce GTP. Nevertheless, the lack of clear specificity for either GDP or ADP exhibited by the kinases catalyzing the downstream reactions suggests that some nucleotide flexibility could be permitted.

### Introduction of an ATP-dependent glucokinase in *R. cellulolyticum*

To challenge the hypothesized nucleotide flexibility of the CCM in *R. cellulolyticum*, its native GTP-dependent hexokinase was replaced by the ATP-dependent glucokinase (GLK)^20^ from *E. coli*. The strong preference of GLK for ATP was verified as a prerequisite (Fig. 3 and Fig S3A), though some weak activity was detected with GTP for the enzyme purified from an overproducing *E. coli* strain (Fig. S3A). Furthermore, in the same experimental conditions and at saturating concentrations of their respective preferred NTP, the *R. cellulolyticum* HK (*k*_cat_ = 8,804 ± 177 min^-1^ and *K*_m_ = 0.21 ± 0.02 mM)^15^ and the *E. coli* GLK (*k*_cat_ = 13,088 ± 511 min^−1^ and *K*_m_ = 0.21 ± 0.04 mM) (Fig. S3B) display similar kinetic parameters for glucose. However, the *K*_m_ value for GTP of HK (0.40 ± 0.07 mM)^15^ is 6-fold lower than that of GLK for ATP (2.47 ± 0.39 mM). A gene encoding the *E. coli* GLK and adapted to *R. cellulolyticum* codon bias was synthesized and cloned into the plasmid pSOS956^21^, downstream of a constitutive promoter (weakened P_*thl*_ promoter^22^), thereby generating the plasmid pSOS-GLK. The *R. cellulolyticum* MTL3221 mutant strain, in which the gene encoding HK was formerly inactivated^15^ and renamed hereafter Δ*hk*, was transformed with an “empty” pSOS956 plasmid (pSOS-0), the pSOS-GLK above-mentioned and a pSOS956 plasmid harboring the gene encoding the endogenous HK (pSOS-HK).

**Fig. 4:**
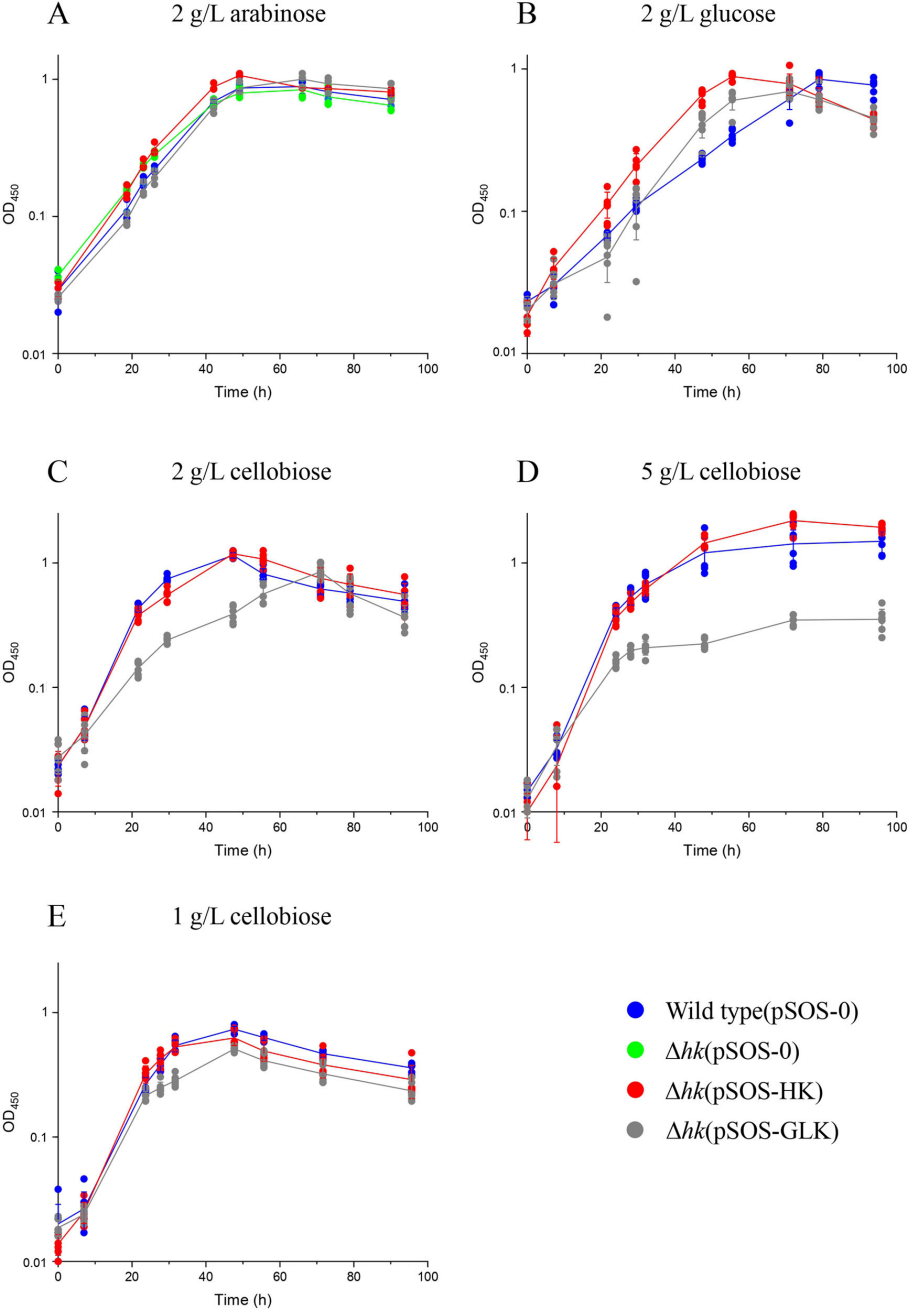
Growth of the control and mutant strains of *R. cellulolyticum.* Growth of *R. cellulolyticum* on **A** 2 g/L arabinose, **B** 2 g/L glucose, **C** 2 g/L cellobiose, **D** 5 g/L cellobiose and E) 1 g/L cellobiose. The growth substrate and its initial concentration are indicated on top of each graph. The cultures were inoculated (1/20) with arabinose (2 g/L)-grown precultures for (**A**), with glucose (2 g/L)-grown precultures for (**B**), and with cellobiose (2 g/L)-grown precultures for **C**, **D,** and **E**. The growths of wild-type(pSOS-0) (blue), Δ*hk*(pSOS-0) (green), Δ*hk*(pSOS-HK) (red) and Δ*hk*(pSOS-GLK) (gray) are shown. The growth was monitored at 450 nm. Since Δ*hk*(pSOS-0) cannot grow on glucose and cellobiose-based medium^14^ (Fig. S6), the growth of this specific mutant strain in these experimental conditions (same sugar used in the preculture) could only be monitored on arabinose-based medium (**A**). The data show the means of three biological replicates for (**A**) and six biological replicates for (**B**, **C**, **D**, and **E**). Bars indicate the standard deviations.

The three strains displayed growths on 2 g/L arabinose (whose catabolism does not require any hexose kinase) similar to that of the wild-type strain transformed with pSOS-0 (WT(pSOS-0)) (Fig. 4A). On glucose-based medium (2 g/L) the Δ*hk*(pSOS-0) strain is unable to grow^15^, whereas, WT(pSOS-0), Δ*hk*(pSOS-HK) and Δ*hk*(pSOS-GLK) strains exhibited similar growth rates, with doubling times of 11 ± 0.3 h, 9.6 ± 0.9 h, and 9.3 ± 1.2 h, respectively (Fig. 4B), as well as similar release of fermentation products (Fig. S4A-D). This indicates that the ATP-dependent GLK can substitute for the GTP-dependent HK, and that the CCM can efficiently handle the excess ADP generated by the *E. coli* enzyme. In contrast, on the disaccharide cellobiose at 2 g/L (Fig. 4C and Fig. S4E-H), the Δ*hk*(pSOS-GLK) strain exhibited impaired growth, a slower consumption of cellobiose and a delayed release of fermentation products, compared to that of the Δ*hk*(pSOS-HK) strain. Its generation time (9.3 ± 1.2 h) is doubled compared to that of WT(pSOS-0) and Δ*hk*(pSOS-HK) strains (4.6–4.8 h), and its final biomass is reduced by 30%. Interestingly, the growth of the strain Δ*hk*(pSOS-GLK) was even more impacted on 5 g/L cellobiose (Fig. 4D and Fig. S4I-L), with final biomass reduced by 83% compared to that of the strain Δ*hk*(pSOS-HK). In contrast, at low cellobiose concentration (1 g/L), both strains Δ*hk*(pSOS-GLK) and Δ*hk*(pSOS-HK) display rather similar growth parameters (Fig. 4E), and release of fermentation products (Fig. S4M-P).

### Introduction of the GTP-dependent hexokinase in *E. coli*

**Fig. 5:**
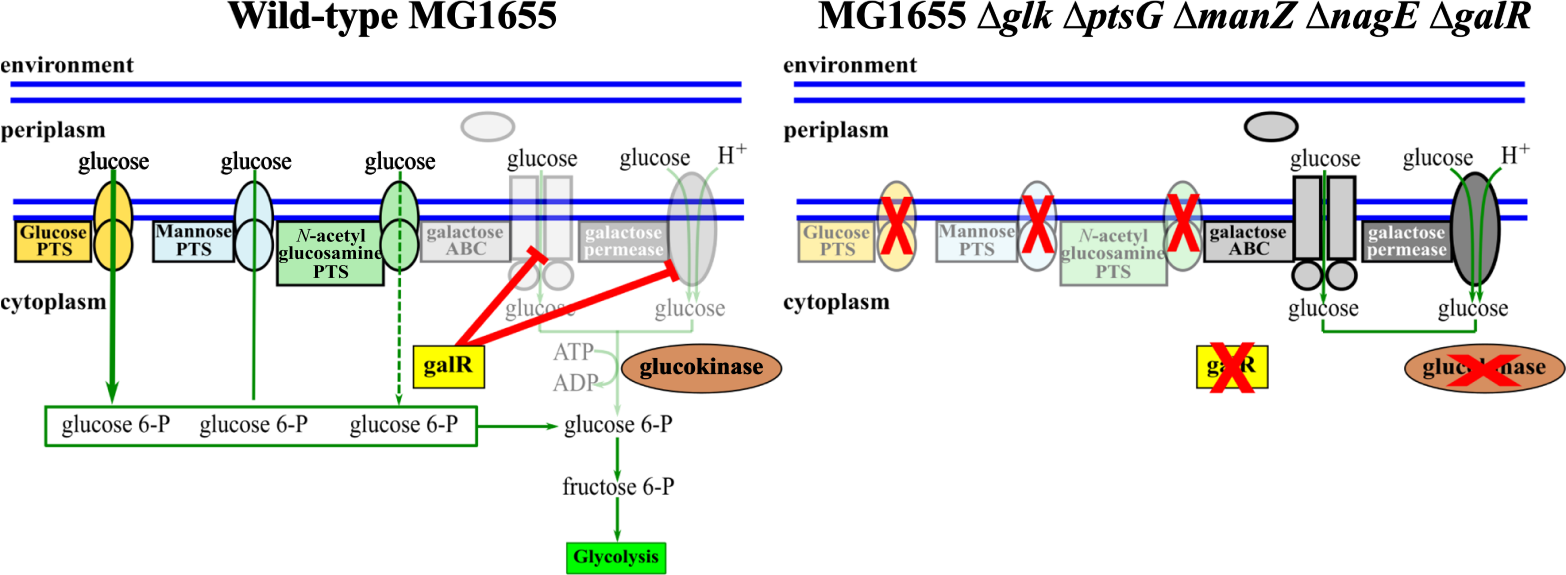
Schematic representation of the strategy used to engineer the glucose^-^ strain of MG1655. The left panel shows the glucose uptake systems available in the wild-type strain, which include the glucose PTS (orange), mannose PTS (light blue), and N-acetyl glucosamine PTS (light green) systems, which import glucose in a phosphorylated form. Two other uptake systems also known to import glucose, the galactose ABC-transporter, and the galactose permease (in gray) are also shown, but are partly erased, since these systems are very weakly synthesized, due to a strong repression (symbolized as thick red lines) of their encoding genes in the absence of galactose, by the regulator galR, shown as a yellow square. The glucokinase is symbolized by a brown oval. The right panel shows the strategy used to create a modified strain that can import glucose but exclusively in a non-phosphorylated form, and incapable to metabolize the imported glucose. Deletion of the genes, *ptsG*, *manZ*, and *nagE* allowed the inactivation of the glucose PTS, mannose PTS, and N-acetyl glucosamine PTS systems, respectively. Deletion of the gene *glk* encoding the endogenous glucokinase, inactivates the first step of glycolysis. Deleting the *galR* gene, which encodes the galR repressor, enables the constitutive production of both the galactose permease and the galactose ABC-transporter. Red crosses symbolize the inactivated systems, regulator, or enzyme in the modified strain. Horizontal parallel blue thick lines symbolize the inner and outer membranes. The thickness of the vertical green arrows crossing the uptake systems in the inner membrane denotes the glucose flux across these systems (left panel).

The reverse approach was applied to investigate if the native ATP-dependent GLK in *E. coli* could be replaced by the GTP-dependent HK from *R. cellulolyticum*. A prerequisite was the construction using the Keio collection^23^ of an *E. coli* MG1655 strain retaining its ability to uptake glucose but unable to catabolize this hexose in the absence of GLK (*E. coli* MG1655 Δ*glk* Δ*ptsG* Δ*manZ* Δ*nagE* Δ*galR* mutant strain, hereafter referred to as glucose^−^, Fig. 5).

In this mutant strain, the genes encoding components of three PTS systems known to uptake glucose (*ptsG, manZ, nagE*) were deleted along with the gene encoding the endogenous glucokinase^24^. We subsequently deleted the gene encoding the regulator GalR, which, in the absence of galactose, represses the expression of the genes encoding the galactose ABC-transporter and the galactose permease, since both can import glucose^25^ in a non-phosphorylated form. Thus, the resulting strain retains the ability to uptake glucose via the galactose transporters which are constitutively synthesized but cannot catabolize the hexose due to *glk* deletion. A gene adapted to *E. coli* codon bias encoding the *R. cellulolyticum* HK was then synthesized and cloned into the vector pJRD300^26^, downstream of the P_*pdc*_ promoter from *Zymomonas mobilis*^27^. This promoter was formerly shown to allow continuous but moderate expression of its associated gene in *E. coli*^26^. The resulting vector (pJRD-HK) and similar vectors containing no gene (pJRD-0) or the gene encoding the endogenous *E. coli* glucokinase (pJRD-GLK) were used to transform theglucose^-^ strain.

**Fig. 6:**
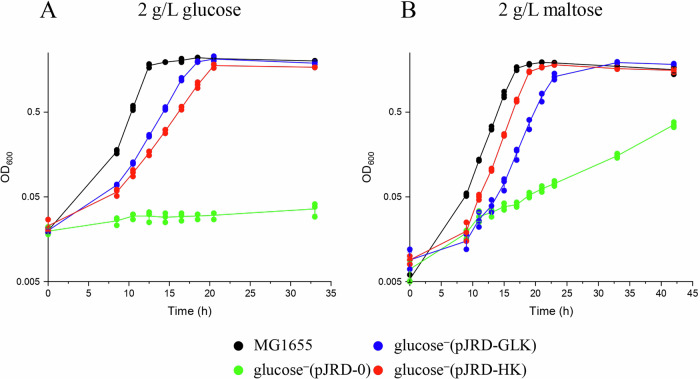
Growth of the control and mutant strains of *E. coli.* Growth of *E. coli* on **A** glucose, **B** maltose (2 g/L). Cultures were inoculated (1/500) with LB-grown precultures. The growths of wild-type MG1655 (black), glucose^-^ (pJRD-0) (green), glucose^-^ (pJRD-HK) (red) and glucose^-^ (pJRD-GLK) (blue) are shown. The growth was monitored at 600 nm. The growth substrate is indicated at the top of each graph. The data show the means of three independent experiments, and bars indicate the standard deviations.

As expected, the glucose^−^ (pJRD-0) strain displayed no growth in the M9 medium supplemented with glucose (Figs. 6A and S5A), whereas strains transformed with either the pJRD-GLK or the pJRD-HK exhibited rapid growth in this medium. The replacement of GLK by HK in the glucose^-^ (pJRD-HK) strain induced only a slightly impaired growth and glucose consumption (Figs. 6A and S5A) compared to that of the glucose^−^ (pJRD-GLK) strain, reflected by their doubling times of 147 ± 10.4 min and 109 ± 3.5 min, respectively. However, the rather robust growth observed for the glucose^−^ (pJRD-HK) strain, indicates that the GTP concentration is sufficient for HK to be operational and that the CCM in *E. coli* can tolerate a GTP-consuming (and GDP-producing) key metabolic kinase.

Since the *E. coli* MG1655 strain cannot uptake and utilize cellobiose unless extensive engineering^26^, we selected the disaccharide maltose to further explore the metabolic capabilities of the aforementioned *E. coli* strains. Maltose is imported through a maltodextrin ABC-transporter and is then catabolized to glucose and glucose-1-P by the combined actions of cytoplasmic amylomaltase (MalQ), maltodextrin phosphorylase (MalP), and maltodextrin glucosidase (MalZ)^28^. The expression of all *mal* genes is under the positive control of the regulator MalT. As shown in Fig. 6B, the glucose^−^ (pJRD-0) strain exhibited a very slow growth on maltose, contrary to the glucose^−^ (pJRD-HK) strain displaying a rapid growth on this disaccharide, with a doubling time (88.7 ± 1.0 min) very similar to that of the wild-type MG1655 strain (92 ± 4.0 min), but also slightly shorter than that of the glucose^-^ (pJRD-GLK) strain (105.4 ± 6.7 min) (Fig. 6B and S5B). Plasmid-based overexpression of the *glk* gene was previously shown to reduce the expression of the *mal* genes and impair the growth of maltose^29^. It was conjectured that overproduced glucokinase could directly interact with MalT, and inhibit this regulator. The slightly faster growth of strain glucose^−^(pJRD-HK) compared to strain glucose^−^(pJRD-GLK) on maltose supports this hypothesis, as HK and GLK share no significant sequence similarity. Therefore, as observed for the glucose-based medium, the replacement of the ATP-dependent GLK by the GTP-dependent HK allows robust growth in the maltose-containing medium, thus confirming the CCM in *E. coli* can handle a GDP-producing metabolic kinase.

### Quantification of the purine nucleotide pools in wild-type *E. coli* and *R. cellulolyticum*

Altogether, our data indicate that the CCM in *R. cellulolyticum* is essentially GTP-driven, unlike *E. coli*. After quantification of the purine nucleotides in both bacteria (Table 2) as formerly described^30^, a typical ATP/GTP ratio of 2.4 ± 0.19 is obtained for *E. coli* grown aerobically^30,31^ but this ratio is significantly reduced (*P* < 0.01) to 1.2 ± 0.44 for *R. cellulolyticum*. This observation leads us to propose that GTP also constitutes an important energy currency in the anaerobic bacterium.

**Table 2:** Amounts of adenosine and guanosine phosphates in crude extracts of wild-type *E. coli* and *R. cellulolyticum*

Bacterial species	ADP (nmol)	ATP (nmol)	GDP (nmol)	GTP (nmol)	ATP/GTP
*E. coli* MG1655^a^	7.07 ± 1.92	11.17 ± 2.13	6.11 ± 1.55	4.6 ± 0.89	2.43 ± 0.19
*R. cellulolyticum*^b^	3.95 ± 0.68	1.24 ± 0.57	1.23 ± 0.45	1.04 ± 0.35	1.22 ± 0.44

^a^Means of three biological replicates (10 mL of suspension containing 2.0 ± 0.1 × 10^9^ cells), and two technical replicates for each biological sample. *E. coli* was grown aerobically in M9 medium supplemented with 2 g/L of glucose.

^b^Means of three biological replicates (10 mL of suspension containing 2.0 ± 0.3 × 10^9^ cells) and three technical replicates for each biological sample. *R. cellulolyticum* was grown anaerobically in basal medium supplemented with 2 g/L of cellobiose.

## Discussion

ATP is generally recognized as the energy currency of living cells^32^, while GTP is considered to play a pivotal role in signaling pathways and protein synthesis. However, hydrolysis of either ATP or GTP yields the same standard Gibbs energy (ΔG^0^’) of –30.5 kJ/mol, and the reasons why ATP was selected as the primary energy carrier by living organisms remain uncertain. In heterotrophs, a major source of energy for cell growth and development is provided by the CCM^4^, but our study shows that contrary to most other known organisms, this crucial pathway appears to be essentially GTP-driven in *R. cellulolyticum*. Consistently, we observed that, unlike *E. coli*, GTP is almost as abundant as ATP in the cellulolytic bacterium, and also probably serves as an important energy currency.

Our present data do not provide a clear explanation for the reliance of its CCM on GTP. However, it is worth noting that a few sugar kinases involved in the CCM exhibiting a preference for GTP over ATP were previously documented, predominantly in thermophilic microorganisms^11,33,34^. The other kinases of their CCM were generally not examined, but one could speculate that a putative increased half-life of GTP compared to ATP could explain the selection of GTP for key metabolic reactions by some thermophiles. Consistently, other studies focusing on the stability of metal ion complexes with nucleoside triphosphate in aqueous solution also trend toward an increased stability of GTP^35,36^. The cellulosome-producing bacterium *R. cellulolyticum* is mesophilic, but it grows slowly (doubling time around 24 h) when crystalline cellulose is the sole carbon source^37^, due to the slow extracellular conversion of cellulose into fermentable cellodextrins^38–40^. Previous flux analyses have shown that its metabolism is well adapted or “even restricted” to the low carbon flow generated by its “cellulolytic lifestyle”^41^. In this context of low-rate metabolism, one could hypothesize that the selection of an NTP with extended half-life as an energy carrier might also be advantageous.

Another particularity of the *R. cellulolyticum* CCM, unveiled in the present study, is the lack of a marked preference for either GDP or ADP exhibited by the kinases catalyzing the downstream (forward glycolytic) reactions. This observation led us to question the NTP/NDP flexibility of the CCM in *R. cellulolyticum* in vivo by replacing the endogenous GTP-dependent HK with the ATP-dependent GLK from *E. coli*^20^. This replacement had no significant effect on glucose utilization by the bacterium, thus confirming that the ADP generated by GLK was efficiently processed by the NDP-consuming kinases catalyzing the downstream reactions. However, significant but impaired growth was observed for the GLK-producing strain on the disaccharide cellobiose at 2 g/L. This phenomenon was amplified when the initial cellobiose concentration was raised to 5 g/L, but almost abolished when the concentration of cellobiose was reduced to 1 g/L. After uptake through an ABC-transporter^42^, cellobiose is converted into α-glucose 1-P and glucose by a specific cellobiose phosphorylase, for which glucose acts as a strong competitive inhibitor (*K*_i_ = 0.68 mM)^15^. We hypothesize that the impaired growth observed for Δ*hk*(pSOS-GLK) is likely because the glucokinase in this cellular context does not consume the released glucose fast enough compared to the endogenous hexokinase, especially at the highest cellobiose concentration (5 g/L) where growth is most impaired. As shown in Table 2, the amount of ATP measured in *R. cellulolyticum* extracts is ninefold lower compared to that in *E. coli*. Since both bacterial cells have similar volumes (around 1.5 µm^3^)^43^ it can be extrapolated that the cytoplasmic concentration of ATP in the anaerobic bacterium is ~0.4 mM, a value sixfold lower than the *K*_m_ of GLK for ATP. Therefore, unlike the *R. cellulolyticum* enzyme, the impaired activity of GLK due to low cellular ATP fails to maintain cytoplasmic glucose at sub-inhibitory concentrations for the cellobiose phosphorylase, thus limiting the rate of cellobiose breakdown^15^. This phenomenon was not observed when glucose was the sole carbon source, but the doubling times of all strains were around 9.5–11 h. This suggests that even if the phosphorylation of glucose is slower in the Δ*hk*(pSOS-GLK) strain, another step in the glucose assimilation, possibly the uptake, remains limiting. Nevertheless, the significant growth observed for the GLK-producing strain on both substrates indicates that the CCM in *R. cellulolyticum* is flexible regarding nucleotides.

The reverse approach was applied to *E. coli* and engendered an HK-producing strain displaying robust growth on both glucose- and maltose-based media. An extrapolation based on the data in Table 2 provides an estimate of the intracellular concentration of GTP in *E. coli* around 1.5 mM, whereas the *K*_m_ value of HK for GTP is only 0.4 mM. Thus, HK in *E. coli* has enough GTP to efficiently phosphorylate glucose, in contrast to GLK in *R. cellulolyticum*. Indeed, these results also indicate that *E. coli* can efficiently cope with the additional GDP flux generated by the heterologous enzyme on both substrates. This unanticipated flexibility may suggest that similarly to *R. cellulolyticum*, the NTP-producing kinases involved in the downstream reactions in *E. coli* may also effectively function with GDP as the co-substrate, although this was never reported. It is more likely that the nucleoside diphosphate kinase (NDK), which catalyzes the reversible phosphorylation of NDP using ATP as the phosphate donor^44^, and/or the polyphosphate kinase^45^ (PPK) of *E. coli*, could come into play to handle this additional GDP flow. Interestingly, the ubiquitous genes *ndk* and *ppk*, encoding NDK and PPK, respectively, could not be found in the genome of *R. cellulolyticum* and related cellulolytic clostridia. In this context, the kinases catalyzing the downstream reactions in *R. cellulolyticum* which lack a preference for either GDP or ADP, especially the reversible enzymes PGK and AK, could play an important role in maintaining the NTP pool homeostasis.

In conclusion, our data indicate that the CCM in heterotrophic prokaryotes can significantly diverge from canonical models, and display unexpected nucleotide flexibility despite its crucial importance as a major energy source for the cell.

## Methods

### Strains and media

Wild-type *R. cellulolyticum* (ATCC 35319) and Δ*hk* mutant strains hosting pSOS956 derivates (pSOS-0, pSOS-GLK, or pSOS-HK) were grown at 32 °C in basal medium^37^ supplemented with 2 g/L of either arabinose, glucose or 1, 2 and 5 g/L cellobiose, and 2.5 µg/mL thiamphenicol. *E. coli* BL21(DE3) strain was used for protein production and grown at 37 °C in lysogeny broth supplemented with 50 µg/mL kanamycin. The *E. coli* NEB5α strain was used for cloning purposes. The wild-type MG1655 strain and the MG1655 Δ*glk* Δ*ptsG* Δ*manZ* Δ*nagE* Δ*galR* mutant strain (also termed glucose^−^) carrying the pJRD300 derivates (pJRD-0, pJRD-GLK or pJRD-HK) were grown at 37 °C in M9 synthetic medium supplemented 2 g/L glucose or maltose, and 50 µg/mL kanamycin where applicable. Growth was followed by optical density measurements at 450 nm (*R. cellulolyticum*) or 600 nm (*E. coli*).

### Generation of the MG1655 Δ*glk* Δ*ptsG* Δ*manZ* Δ*nagE* Δ*galR* mutant strain, and construction of pSOS956, pJRD300, pET28a, and pET22b+ derivates

P1 *vir* phage was used to prepare lysates from the donor strains. The deletion mutants were prepared by transducing the recipient strain with the corresponding Keio (FRT)kan deletion and then curing the kanamycin resistance using the pCP20 plasmid^23,46^. The genomic DNA of the MG1655 Δ*glk* Δ*ptsG* Δ*manZ* Δ*nagE* Δ*galR* mutant strain was verified by sequencing (Novogene, Cambridge, UK).

The pSOS-0 and pSOS-HK were formerly made available^15^. To construct pSOS-GLK, a gene encoding *E. coli* (MG1655) glucokinase adapted to *R. cellulolyticum* codon bias was designed (see supplementary information) and synthesized (GenScript Biotech, Rijswijk, the Netherlands). The synthetic gene was amplified by PCR using the primer pair GlkBamH1f/GlkNar1R (see Table S1), and cloned in pSOS956 at the BamHI/EheI sites, downstream a weakened P_*thl*_ promoter.

pJRD-0 was obtained by digestion of pJRD300-RA^26^ with EcoR1. For pJRD-GLK, the *glk* gene from MG1655 strain was amplified using the primer pair glkNde1F/glkXba1R, and cloned in pJRD300-RA^26^ at Nde1/Xba1 sites, thereby generating pJRD-GLK. The vector pJRD-HK was constructed as follows: a gene encoding the *R. cellulolyticum* hexokinase adapted to *E. coli* codon bias (see supplementary information), was designed, synthesized (GenScript Biotech), amplified using the primer pair hexo3221Nde1F/hexo3221Xba1R and cloned in pJRD-RA at NdeI/XbaI sites, thereby generating the pJRD-HK.

The genes encoding XK, PGK, PK, and AK were amplified from genomic *R. cellulolyticum* DNA using the primer pairs Ccel_3431F/Ccel_3431R, Ccel_2260F/Ccel_2260R, Ccel_2569F/Ccel_2569R and Ccel_2136F/Ccel2136R, respectively. The resulting amplicons were digested by NcoI/XhoI, and cloned in NcoI-XhoI-linearized pET28a (Novagen, Madison, WI), thereby leading to pET28a-XK, pET28a-PGK, pET28a-PK and pET28a-AK, which encode C-terminal His-tagged xylulokinase, phosphoglycerate kinase, pyruvate kinase and acetate kinase, respectively. The *glk* gene encoding the glucokinase in *E. coli* was amplified from MG1655 strain genomic DNA using the primer pair glkF/glkR, and cloned in pET22b+ (Novagen) at NdeI/XhoI sites, thereby leading to pET22b+-GLK which encodes C-terminal His tagged glucokinase.

### Production in *E. coli* and purification of the selected metabolic kinases

The BL21(DE3) strains carrying the pET28 derivates were grown in 2 to 5 flasks containing 700 mL of LB supplemented with glycerol (12 g/L) and 50 µg/mL kanamycin (pET28a derivates) or 200 µg/mL ampicillin (pET22b+ derivate) at 37°C until OD_600_ ≈ 1.5. Induction of the expression was performed overnight at 20–22 °C using 200 µM of isopropyl-thio-β-D-galactoside. Cells were harvested by centrifugation (3000 *g*, 10 min, 4 °C), resuspended in 30–80 mL of 30 mM Tris-HCl pH 8.0 (THC), 5 mM imidazole supplemented with a few mg of DNaseI (Roche, Basel, Switzerland), and broken in a French press (Stansted Fluid Power Ltd, Harlow, UK). The extract was centrifuged (15,000 *g*, 20 min at 4 °C), and the supernatant was loaded on 2–4 mL of HisPurTM Ni-NTA resin (Thermo Scientific, Rockford, IL) equilibrated in the same buffer. Elution of His-tagged proteins of interest was performed using 100 mM imidazole in THC. The purification was achieved on a 1-mL HiTrap Q HP fast flow column (Cytiva, Marlborough, MA) equilibrated in THC. Elution was performed using a linear gradient of 0–500 mM NaCl in THC. In the case of the PGK, another chromatography using gel filtration was required to achieve its purification to homogeneity. The fraction of interest was loaded on a HiLoad Superdex 200 (Cytiva) equilibrated in 30 mM Tris-HcCl pH 8.0, 0.15 M NaCl at 2 mL/min. The purified proteins were dialyzed and concentrated by ultrafiltration in Vivaspin20 (cut-off 10 kDa, Sartorius, Göttingen, Germany) against 10 mM Tris-HCl pH 8.0, and stored at −80 °C. The concentration of the proteins was estimated by absorbance at 280 nm using the program ProtParam tool (www.expasy.org/tools/protparam.html).

### Characterization of selected metabolic kinases

The kinetic parameters of XK for the NTP were determined by High-Pressure Liquid Chromatography on a (300 ×7.8 mm) Aminex HPX-87H column (Bio-rad, Hercules, CA) coupled with Refractive Index detector (Iota, Marseille, France) (HPLC-RI)^47^: the enzyme (10 nM) was incubated at 37 °C in 25 mM HEPES buffer pH 7.0, 20 mM MgCl_2_ containing 2.5 mM xylulose (Sigma-Aldrich, ST Louis, MO), and either GTP (at final concentrations ranging from 0.125 to 25 mM) or ATP (at final concentrations ranging from 0.75 to 25 mM). One hundred µL-aliquots were pipetted at 5 and 15 min and mixed with 25 µL of 25 mM H_2_SO_4_. The samples were analyzed by HPLC-RI (flow rate was 0.6 mL/min and 55°C): xylulose, ATP, and GDP were eluted using 5 mM H_2_SO_4_ for 20 min. Injections of xylulose, ATP, and GDP at known concentrations were used to determine the activity, and the software Origin 2019b was used to establish the *k*_cat_ and *K*_m_ values based on a Michaelis-Menten model. The kinetic parameters of XK for xylulose were determined similarly, in the presence of either 10 mM GTP or 25 mM ATP, and with xylulose final concentrations ranging from 0.25 to 20 mM.

The kinetic parameters of the PGK on NDP were determined in coupled assays with commercial GAPDH (Roche) at 37 °C by following for 5 min the absorbance at 340 nm of the NAD^+^ reduction (forward glycolytic reaction)^10^. The assay was performed in 50 mM potassium phosphate pH 7.0, 15 mM cysteine, 10 mM MgCl_2_, 1.1 mM NAD^+^, 3 mM glyceraldehyde-3-P 80 nM of PGK, and either GDP or ADP at concentrations ranging from 25 µM to 1 mM. The reaction was started by adding 7.5 µg of GAPDH. The reverse reaction was also monitored in 50 mM potassium phosphate pH 7.0, 15 mM cysteine, 10 mM MgCl_2_, containing 0.3 mM NADH, 4 mM (for ATP experiment) or 25 mM (GTP experiment) 3-phosphoglycerate, 7.5 µg of GAPDH, and either GTP or ATP at concentrations ranging from 100 µM to 10 mM. The reaction was started by adding 33.3 nM PGK (ATP) or 80 nM PGK (GTP). The *k*_cat_ and *K*_m_ values of PGK for 3-phosphoglycerate were established similarly in the presence of 10 mM ATP or 25 mM GTP, and variable concentrations of 3-phosphoglycerate (0.1–10 mM).

The kinetic parameters of PK for NDP were determined by HPLC-RI as described above. The enzyme (50 nM for ADP, 25-100 nM for GDP) was incubated at 37°C in 25 mM HEPES (pH 7.0), 20 mM MgCl_2_, 30 mM KCl, 10 mM PEP, FBP at 0, 0.5, 3 or 10 mM, and variable concentrations of NDP (0.25 to 10 mM). One hundred µL-aliquots were pipetted at 5 min and mixed with 25 µL of 25 mM H_2_SO_4_. The samples were analyzed by HPLC-RI: pyruvate was eluted using 5 mM H_2_SO_4_ for 20 min. Injections of pyruvate at known concentrations were used to determine the activity. The *k*_cat_ and *K*_m_ values of PK pour PEP were determined in similar experimental conditions except that the concentration of NDP was kept constant at 5 mM, and the concentration of PEP varied from 0.25 to 25 mM.

The kinetic parameters of AK for NDP were determined by HPLC-RI as described above. The enzyme (5 or 8 nM) was incubated at 37 °C in 25 mM HEPES (pH 7.0), 20 mM MgCl_2_, and 5 mM acetyl-P (Sigma-Aldrich) containing variable concentrations of NDP (0.25 to 10 mM). 100-µL-aliquots were pipetted at 5 min and mixed with 25 µL of 25 mM H_2_SO_4_. The release of acetate or ATP was monitored. Injections of acetate and ATP at known concentrations were used to determine the activity. The kinetic parameters of AK for acetyl-P were established similarly except that NDP concentration was constant at 10 mM, and the concentration of acetyl-P ranged from 0.25 to 10 mM. The reverse reaction was also monitored. The *k*_cat_ and *K*_m_ values of AK for ATP were determined in the same buffer containing 10 mM acetate and variable concentrations of ATP (ranging from 0.25 to 10 mM). The enzyme concentration was 6 µM. The kinetic parameters for acetate were determined similarly, at the same AK concentration of 6 µM but in the presence of 10 mM ATP, and variable concentrations of acetate (ranging from 0.25 to 10 mM).

The *k*_cat_ and *K*_m_ values of *E. coli* glucokinase for NTP were determined by HPLC-RI as follows: The enzyme (50–100 nM) was incubated at 37 °C in 25 mM HEPES (pH 7.0), 20 mM MgCl_2_, 5 mM glucose, containing variable concentrations of ATP (0.25-20 mM) or GTP (0.5-40 mM). At specific time points (1 and 2 min for ATP experiments, and 10–80 min for GTP experiments) 100-µL samples were pipetted and mixed immediately with 25 µL of 25 mM H_2_SO_4_. Injections of glucose at known concentrations were performed, and glucose consumption was used to determine the enzymatic activity. The determination of kinetic parameters of the glucokinase for glucose followed a similar protocol, except that the enzyme concentration was adjusted to 10 nM, the concentration of ATP was constant at 10 mM, and glucose concentration varied from 0.05 mM to 20 mM. For glucose concentrations of 50 µM and 100 µM, 100-µL samples were mixed with 25 µL of 0.25 M NaOH and analyzed by high-pressure anion-exchange chromatography coupled with pulsed amperometric detection (HPAEC-PAD, Thermo Fisher Scientific, Waltham, MA), using a PA1 column as formerly described^15^.

### Quantification of ATP, ADP, GTP, and GDP pools in *E. coli* and *R. cellulolyticum*

The concentration of the NTP/NDP was determined essentially as formerly described^30^. Briefly, 10-mL suspensions of cells containing ~2 × 10^9^ cells (counted using a Mallassez cell), were rapidly vacuum filtered on a 0.45 µm filter, which was then immediately plunged into 600 µL of ice-cold 1 M acetic acid, before freezing in liquid nitrogen. Samples were then thawed on ice, and centrifuged for 2 min at 5000 *g* (4 °C). The liquid fraction was collected and frozen again in liquid nitrogen, before lyophilization. The lyophilized material subsequently was dissolved in 250 µL of ice-cold deionized water and centrifuged for 30 min at 4 °C and 14,000 *g*. 200 µl of the supernatant was then mixed with 50 µL of 0.25 M NaOH, before analysis by HPLC using a Dionex IonPac AS11-HC column (4 ×250 mm, Thermo Fisher Scientific) preceded by the corresponding guard column (4 ×50 mm). Detection was performed with a coupled conductivity detector and a UV detector (260 nm). Twenty-five µL of samples were injected into the column. The nucleotides were eluted with the buffers deionized water and 0.1 M NaOH as the eluents A and B, respectively, using the following multistep procedure: first separation gradient (20 min, 90% A + 10% B to 50% A + 50% B), second separation gradient (10 min, 35% A  +  65% B to 100% B), column wash (5 min, 100% B), and subsequent column equilibration (15 min, 90% A + 10% B). The flow was 1 ml/min. ATP, GTP, ADP, and GDP at known concentrations (ranging from 10 to 100 µM) were injected to identify and quantify the nucleotides.

### Analysis of the culture supernatants

For growth of *R. cellulolyticum* on glucose- or cellobiose-based medium, 400-µL samples of the cultures were taken at specific time points and centrifuged for 5 min at 15,000 *g* and 4 °C. The supernatants (200 µL) were subsequently mixed with 50 µL of 25 mM H_2_SO_4_ before analyses by HPLC-RI as mentioned above. Injections of glucose, cellobiose, lactate, pyruvate, formate, and ethanol at known concentrations were used to quantify the sugars and the fermentation products. For aerobic cultures of *E. coli* in M9 medium supplemented with 2 g/L of either glucose or maltose, the same procedure was applied, and injections of glucose and maltose at known concentrations were used to quantify the sugars.

## Supplementary information

Supplementary material

Supplementary Data

Description of Additional Supplementary Materials

## Data Availability

The authors declare that all data supporting the findings of this study are available within the paper, the “Supplementary materials” file, and the “Supplementary data” file (i.e., data underlying Figs. 2, 4, 6, S2, S3, S4, S5, S6, and Table 2).
