# Supplementary material for: A GTP-driven central carbon metabolism in the cellulolytic bacterium Ruminiclostridium cellulolyticum
Source: Commun Biol. 2025 Mar 30;8:523. doi: 10.1038/s42003-025-07971-7 (PMC11955521; doi:10.1038/s42003-025-07971-7)
Supplement: Supplementary file 1 — Supplementary material [file 42003_2025_7971_MOESM1_ESM.pdf]

A GTP-driven central carbon metabolism in the cellulolytic bacterium *Ruminiclostridium cellulolyticum*

Nian Liu[1,2,3], Nicolas Vita[1,2], Marion Holmière[1], Séverine Gagnot[1], Gaël Brasseur[1], Pascale de Philip[1], Sandrine Pagès[1], Stéphanie Perret[1] and Henri-Pierre Fierobe[1]*

Supplementary Materials

Page 2: Sequence of the synthetic genes encoding the hexokinase from *R. cellulolyticum* and the glucokinase from *E. coli*.

Page 3: Table S1 List of primers used.

Page 4: Figure S1 Genetic context of relevant genes.

Page 5: Figure S2 Nonlinear (Michaelis-Menten) regression analysis of the activities of the selected enzymes.

Page 7: Figure S3 Nonlinear (Michaelis-Menten) regression analysis of the activities of *E. coli* glucokinase.

Page 8: Figure S4 Sugar consumption and release of fermentation products by control and mutant strains of *R. cellulolyticum.*

Page 11: Figure S5 Sugar consumption by *E. coli* MG1655 wild-type and glucose$^-$ mutant strains.

Page 12: Figure S6 Growth of the *R. cellulolyticum* mutant strain Δ*hk*(pSOS-0) on various sugars.

Supplementary information 1

Sequence of the synthetic gene encoding the glucokinase (GLK) from *E. coli* and adapted to *R. cellulolyticum* codon bias.

ATGACAAAGTATGCTCTGGTTGGTGATGTTGGTGGTACAAATGCTAGGTTGGCTCTGTGTGATATTG
CTTCAGGAGAAATTAGTCAAGCAAAGACATATTCAGGATTAGATTATCCTAGTCTTGAAGCAGTTA
TTAGAGTATATCTTGAAGAACATAAGGTTGAAGTAAAGGATGGATGTATAGCAATTGCATGTCCTA
TAACTGGTGATTGGGTTGCAATGACAAATCATACATGGGCATTTTCAATTGCAGAAATGAAGAAAA
ATTTAGGATTTAGTCATCTTGAAATTATAAACGATTTTACAGCAGTATCAATGGCAATTCCTATGCT
TAAGAAAGAACATCTTATACAGTTTGGAGGAGCAGAACCTGTTGAAGGTAAACCTATTGCAGTATA
TGGAGCAGGTACAGGATTAGGTGTTGCACATCTTGTTCATGTAGATAAAAGATGGGTTTCACTTCC
TGGAGAAGGTGGACATGTAGATTTTGCACCTAATAGTGAAGAAGAAGCAATTATTTTAGAAATTCT
TAGAGCAGAAATTGGTCATGTTTCAGCAGAAAGAGTATTAAGTGGACCTGGTTTAGTTAACCTTTA
TAGAGCAATTGTAAAAGCAGATAATAGATTACCTGAAAACCTTAAGCCTAAGGATATAACAGAAA
GAGCACTTGCAGATTCATGTACAGATTGTAGAAGAGCACTTAGTTTATTTTGTGTTATAATGGGAA
GATTTGGAGGAAATCTTGCATTAAATCTTGGTACATTTGGAGGAGTTTTTATAGCAGGTGGAATAG
TACCTAGATTTTTAGAATTTTTCAAAGCAAGTGGATTTAGAGCAGCATTTGAAGATAAAGGTAGAT
TTAAAGAATATGTACATGATATACCTGTTTATCTTATTGTACATGATAATCCAGGTTTATTAGGTTC
AGGAGCACATCTTAGGCAGACATTGGGACATATTTTGTAA

Supplementary information 2

Sequence of the synthetic gene encoding the hexokinase (HK) from *R. cellulolyticum* and adapted to *E. coli* codon bias.

ATGGGTAGCAAGCTGGAAATCGTGCAGGATGTTATTAACGCGTTCGAGGTGAACAAAGAAAGCAT
GCTGCGTACCGCGATGCTGTTTAAAGAGACGATGGAGAAGAGCCTGAACGGTGAAAAGACCTGCC
TGAAAATGCTGCCGAGCTACATTGGCAAGCCGACCGGTAAAGAGCAAGGCACCTTCATGACCATC
GATATGGGTGGCACCAACTTTCGTTGCACCAAGTACAAAATTAACAACGGTAACTTCGAGAAGGTT
GGCGAAATCAAGCAGAAACTGATTAACAAGGAGAAAAACTATGACCTGACCAAAAGCGACAGCG
ATGAGAAGCAACTGTTCGGTTTTATGGCGGAATGCATCGGCGAGCTGCTGGAACCGGAGGAAAGC
CTGTACCTGGGTAACACCTTCAGCTTTCCGTGCCGTCAGGAAGGTATCAACGACGCGTATCTGATTC
AATGGACCAAAGAAATCACCACCAGCGGCGTGGTTGGTCAGAACATCAACAAGCTGCTGGAGCAA
AGCCTGAAGGAGAAGAACATTAACGTGAAACCGGTTGCGATCCTGAACGATACCGTGGGTACCCT
GCTGGTTGCGATGTACAGCTATCAGACCGCGGACATCGGCAGCATTATGGGTACCGGCCACAACAC
CTGCTACCTGGAGAACAACCACCCGCTGAACGGTCAAAAGATGATCGTGAACATTGAAAGCGGTA
ACTACAACGTGGGCCTGCCGGTTACCAAATATGATGAGATCATTGACAAGAACAGCCAGATCCCG
GGTGCGCAACTGCTGGAGAAAATGGTTAGCGGTTACTATATGGGCAGCCTGCTGAAGGAAGTTTGC
AAAGATCTGTATAAGAACAACGCGCTGTTCACCAACGAAGACGTGGATATCGACGCGTTCTTTAAC
CAGAACTTCAACGCGCTGATGGTTGAGAACTTTATTCTGTACCCGAGCAACACCAAAGAACAATAT
AAGTGCAGCATCGAGGACGCGGAAATTGTGAAACGTGTTAGCGAGGCGATCCTGAAGCGTACCGT
TCGTCTGGTGGCGGTTAGCCACATGGGTATTCTGTTTCACCAGGAGAACAGCGGCACCAGCGTGAA
CAACGAACACGTTATCGCGATTGATGGTACCATCTACGAAAAAATGCCGAACGCGCCGCAACTGAT
GAAAGAGGCGTTCCGTGAAGCGCTGGGCGACGATGCGAGCAACATCGAGATTCGTCTGGTGAAAG
ACGGTAGCGGTCTGGGTGCGGCGATTGCGGCGGCGTTTGCGGTTACCCAGTAA

Table S1 : list of primers used

Name	Sequence (5'→3')	Target
**Primers used for cloning of the targeted genes in *E. coli* expression vectors pET28a or pET22b**		
Ccel_3431F	TTTTCCATGGCATCTTTTCTTATTGGTATTGATCTAGG	Ccel_3431
Ccel_3431R	TTTTCTCGAGTTTCAATATTGTGCTGAGCTGGTTAA	Ccel_3431
Ccel_2260F	TTTTCCATGGCAAGCATGATGAACAAAAAAAC	Ccel_3221
Ccel_2260R	TTTTCTCGAGTGCTTTTGCGATTATTGAGAA	Ccel_3221
Ccel_2259F	TTTTCCATGGCAAGAAGAACAAAAATTATTTGTACATTG	Ccel_3238
Ccel_2259R	TTTTCTCGAGGTTACATCTTATAACCAGCCC	Ccel_3238
Ccel_2136F	TTTTTTCCATGGCTAAGGTTTTAGTTATAAATGCGGGGAG	Ccel_3429
Ccel_2136R	TTTTTCTCGAGCTTAACCAATCTCACTGTTTCTCTTGC	Ccel_3429
glkF	GGGGGGACATATGACAAAGTATGCATTAGTCGG	*glk E. coli*
glkR	TTTTTTCTCGAGCAGAATGTG ACCTAAGGTCTGGCG	*glk E. coli*
**Primers used for cloning of the targeted gene in *R. cellulolyticum* expression vector pSOS956**		
GlkBamH1f	CTAGGATCCAGAATTTAAAAGGAGGGATTAAAATGACAAAGT ATGCTCTGGTTGGTGATGT	synth[a]. *glk*
GlkNar1R	CATAGTGGCGCCTTACAAAATATGTCCCAATGTC TGCCTAAG	synth. *glk*
**Primers used for cloning in pJRD300 *E. coli* expression vector**		
glkNde1F	GGGGGGACATATGACAAAGTATGCATTAGTCGG	*glk E. coli*
glkXba1R	TTTTTTTCTAGATTACAGAATGTGACCTAAGGTCTGGCG	*glk E. coli*
hexo3221Nde1F	GGGGGGCATATGGGTAGCAAGCTGGAAATCGTGCAG	synth. Ccel_3221
hexo3221Xba1R	TTTTTTTCTAGATTACTGGGTAACCGCAAACGCCGCCGC	synth. Ccel_3221

[a]designates synthetic gene adapted to *R. cellulolyticum* codon bias for *glk*, and synthetic gene adapted to *E. coli* codon bias which encodes the *R. cellulolyticum* hexokinase (gene at locus Ccel_3221).

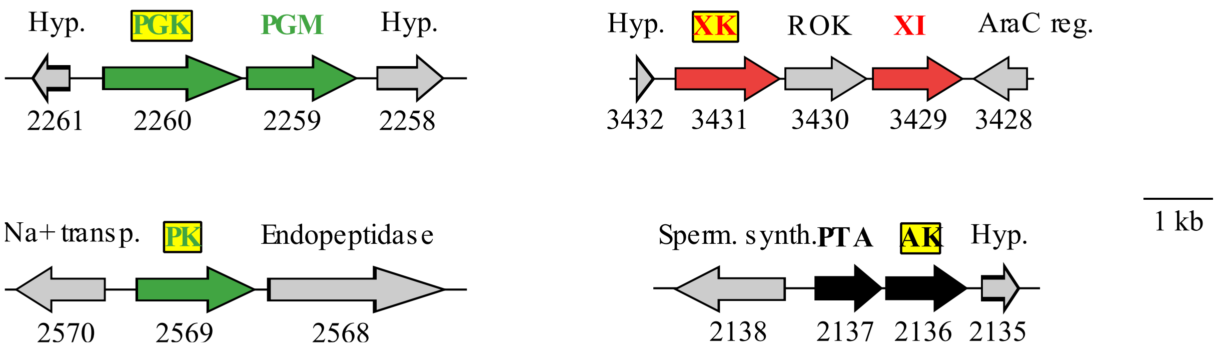

Figure S1. Genetic context of relevant genes. Arrows designate the genes. The predicted encoded proteins and gene loci (e.g., Ccel_2260) are indicated above and below each gene, respectively. PGK and PGM designate phosphoglycerate kinase and phosphoglycerate mutase, respectively. XK and XI designate xylulokinase and xylose isomerase, respectively. PK designates pyruvate kinase. PTA and AK designate phosphotransacetylase and acetate kinase, respectively. ROK indicates Repressor Orf Kinase family protein. Hyp. designates hypothetical protein. AraC reg. indicates AraC family regulator. Na+ transp. designates sodium-dependent transporter, and Sperm. Synth. indicates spermidine synthase. Enzymes shaded in yellow correspond to those characterized in the present study.

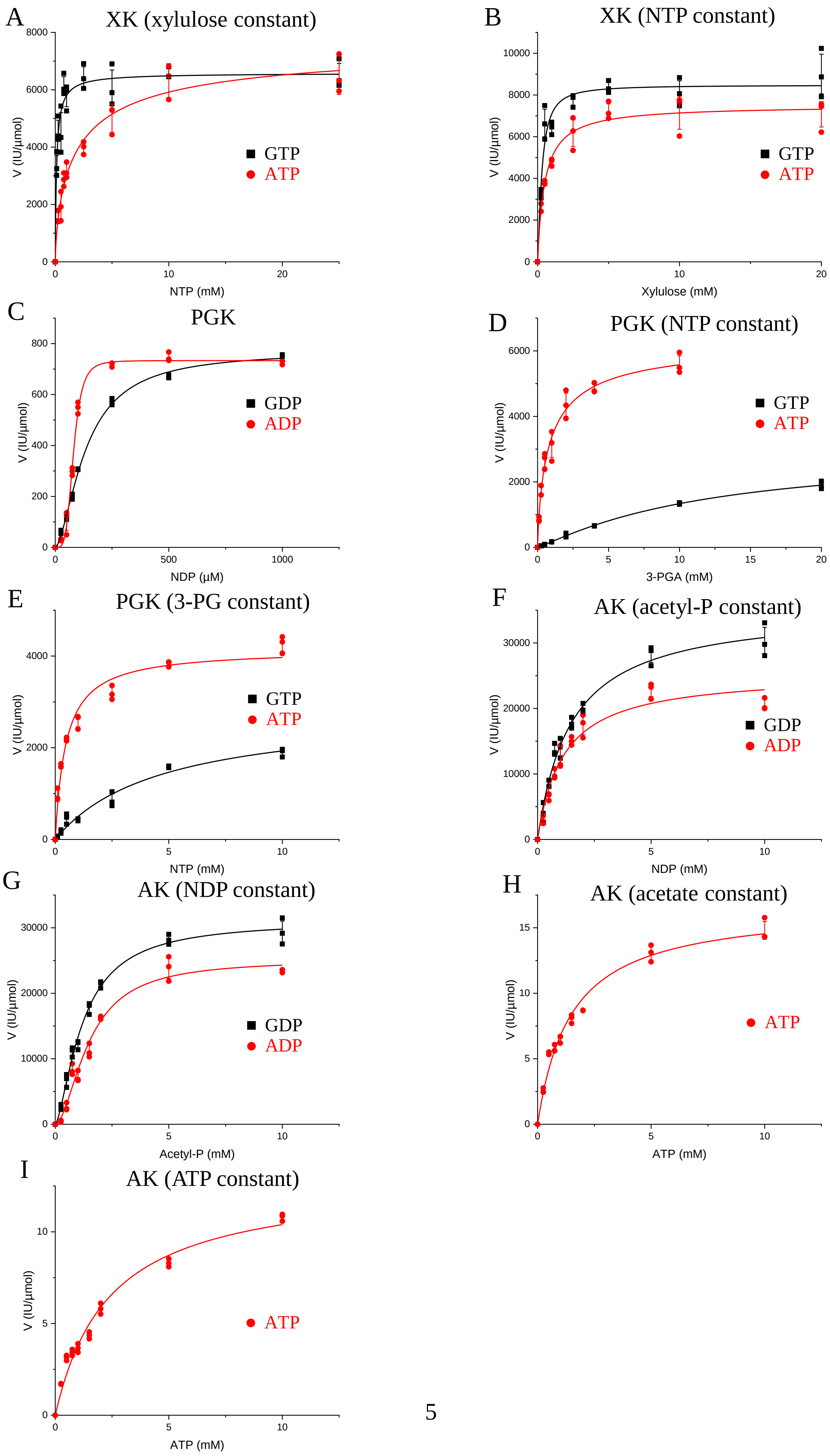
A
XK (xylulose constant)
V (IU/μmol)
NTP (mM)
GTP
ATP
B
XK (NTP constant)
V (IU/μmol)
Xylulose (mM)
GTP
ATP
C
PGK
V (IU/μmol)
NDP (μM)
GDP
ADP
D
PGK (NTP constant)
V (IU/μmol)
3-PGA (mM)
GTP
ATP
E
PGK (3-PG constant)
V (IU/μmol)
NTP (mM)
GTP
ATP
F
AK (acetyl-P constant)
V (IU/μmol)
NDP (mM)
GDP
ADP
G
AK (NDP constant)
V (IU/μmol)
Acetyl-P (mM)
GDP
ADP
H
AK (acetate constant)
V (IU/μmol)
ATP (mM)
ATP
I
AK (ATP constant)
V (IU/μmol)
ATP (mM)
ATP

Figure S2. Nonlinear (Michaelis-Menten) regression analysis of the activities of the selected enzymes. A): analysis of the hydrolytic activity of XK on GTP (black squares) and ATP (red circles) in presence of 2.5 mM xylulose. B) analysis of the phosphorylating activity of XK on xylulose in presence of 10 mM GTP (back squares) or 25 mM ATP (red circles). C): analysis of the phosphorylating activity of PGK on GDP (black square) and ADP (red circles). D): analysis of the phosphorylating activity of PGK on 3-PG in presence of 25 mM GTP (black squares) or 10 mM ATP (red circles). E): analysis of the hydrolysis activity of PGK on GTP (black squares) and ATP (red circles) in presence of either 25 mM or 4 mM 3-PG, for GTP and ATP experiments, respectively. F): analysis of the phosphorylating activity of AK on GDP (black squares) and ADP (red circles) in presence of 5 mM acetyl-P. G): analysis of the hydrolytic activity of AK on acetyl-P in presence of 10 mM GDP (black squares) or 10 mM of ADP (red circles). H): analysis of the hydrolytic activity of AK on ATP in presence of 10 mM acetate. I): analysis of the phosphorylating activity of AK on acetate in presence of 10 mM ATP. The fixed co-substrate is indicated on top of each graph. The data show the means of three independent experiments, and bars indicate the standard deviations. Curves fitting was performed using the Origin 2019b software.

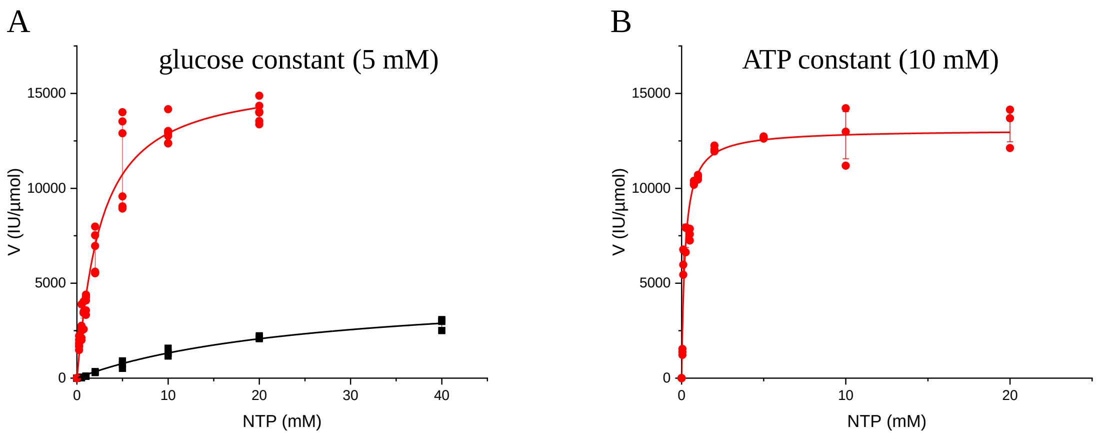

Figure S3. Nonlinear (Michaelis-Menten) regression analysis of the activities of *E. coli* glucokinase. A): analysis of the hydrolytic activity of the glucokinase on ATP (red circles) and GTP (black squares) in presence of 5 mM glucose. B): analysis of the phosphorylating activity of the glucokinase on glucose in presence of 10 mM ATP. The fixed co-substrate concentration is indicated on top of each graph. The data show the means of three independent experiments, except in the case of the activity of the glucokinase on ATP in presence of 5 mM glucose (panel A) for which six experiments were performed. In all cases, bars indicate the standard deviations. Curves fitting was performed using the Origin 2019b software.

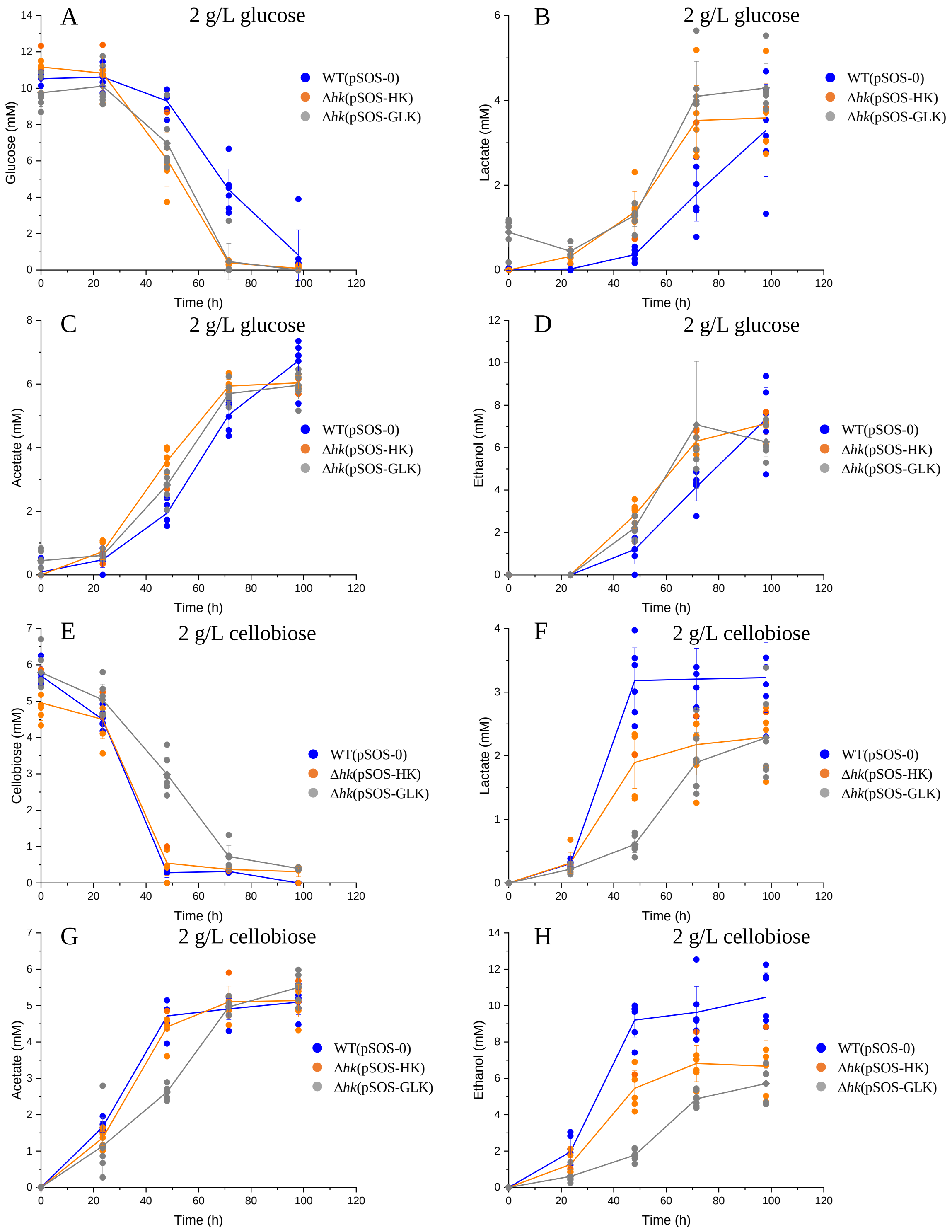

A
2 g/L glucose
Glucose (mM)
Time (h)
B
2 g/L glucose
Lactate (mM)
C
2 g/L glucose
Acetate (mM)
D
2 g/L glucose
Ethanol (mM)
E
2 g/L cellobiose
Cellobiose (mM)
F
2 g/L cellobiose
Lactate (mM)
G
2 g/L cellobiose
Acetate (mM)
H
2 g/L cellobiose
Ethanol (mM)
WT(pSOS-0)
Δhk(pSOS-HK)
Δhk(pSOS-GLK)

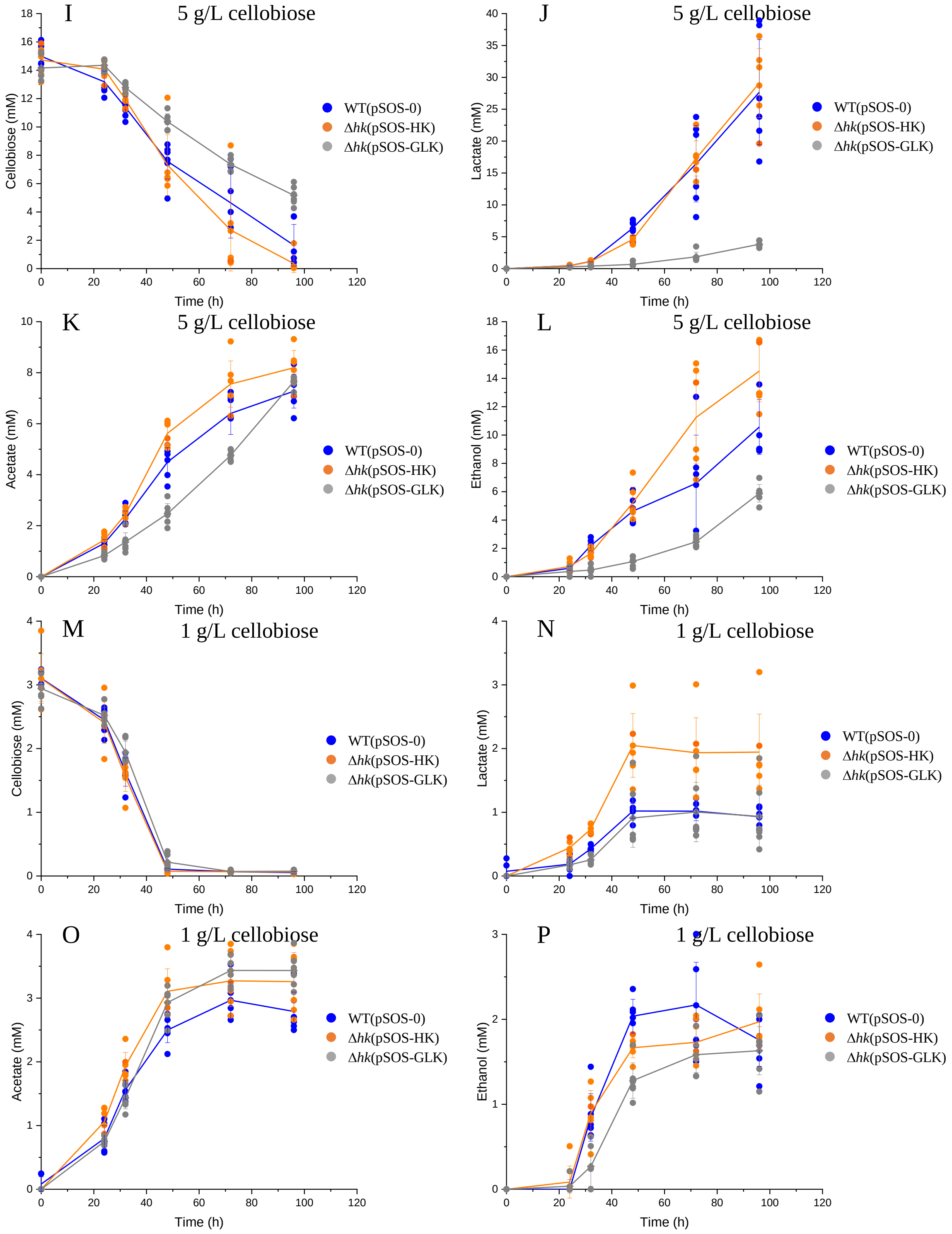

Figure S4. Sugar consumption and release of fermentation products by control and mutant strains of *R. cellulolyticum* on 2 g/L glucose- (A-D), cellobiose-based medium at 2 g/L (E-H), 5 g/L (I-L) and 1 g/L (M-P). Data for WT(pSOS-0) (blue), Δ*hk*(pSOS-HK) (orange) and Δ*hk*(pSOS-GLK) (grey) are shown. A), E), I) and M show glucose and cellobiose consumption. B), F), J) and N) show lactate production; C), G), K) and O) show acetate production; D), H), L) and P) show ethanol production. The data show the means of 6 independent experiments and the bars represent the standard deviations. Samples correspond to the experiments described in

Figures 4B (2 g/L glucose), 4C (2 g/L cellobiose), 4D (5 g/L cellobiose) and 4E (1 g/l cellobiose). The growth substrate and its concentration are indicated at the top of each graph.

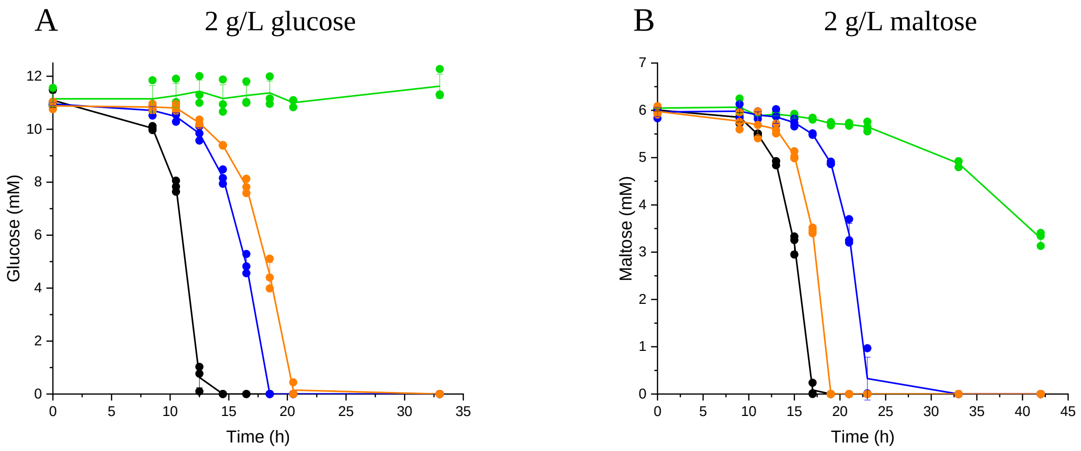

Figure S5. Sugar consumption by *E. coli* MG1655 wild-type and glucose$^-$ mutant strains during growth in M9 medium supplemented with 2 g/L glucose (A) or maltose (B). The data for wild-type MG1655 (black curves), glucose$^-$(pJRD-0) (green curves), glucose$^-$(pJRD-GLK) (blue curves), and glucose$^-$(pJRD-HK) (orange curves) are shown. The data show the means of three biological replicates, and the bars indicate the standard deviations. The data correspond to the experiments described in Figures 6A (glucose) and 6B (maltose). The growth substrate is indicated at the top of each graph.

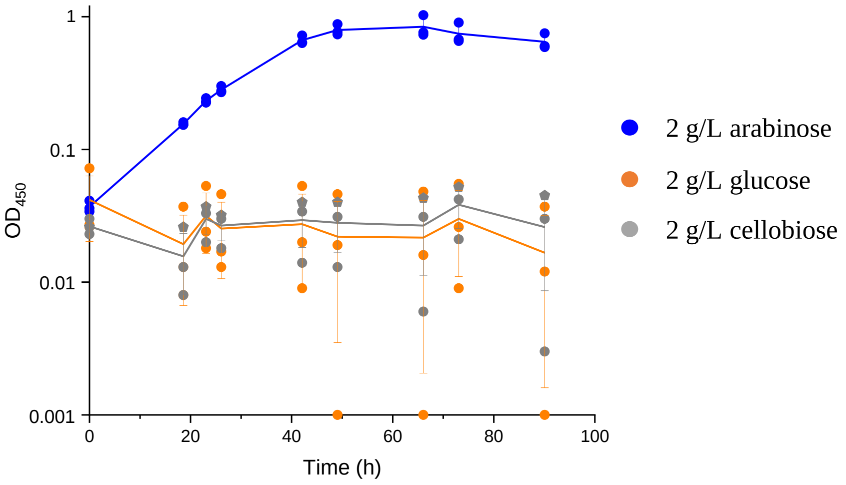

Figure S6. Growth of the *R. cellulolyticum* mutant strain Δ*hk*(pSOS-0) on various sugars. The growth on 2 g/L arabinose (blue circles), 2 g/L glucose (orange circles) and 2 g/L cellobiose (grey circles) based medium are shown. The cultures were inoculated (1/20) with arabinose (2 g/L)-grown precultures. The data show the mean of three independent experiments, and bars represent the standard deviations. In the case of one biological replicate on glucose, the OD_{450} reached 0 at 49.08 h and 90.08 h of incubation (first biological replicate, see Supplementary data). These two individual data points have been placed on the horizontal axis (thus corresponding to an OD_{450} value of 0.001) for viewing in the semi-log plot.
