# Supplementary material for: A GTP-driven central carbon metabolism in the cellulolytic bacterium Ruminiclostridium cellulolyticum
Source: Commun Biol. 2025 Mar 30;8:523. doi: 10.1038/s42003-025-07971-7 (PMC11955521; doi:10.1038/s42003-025-07971-7)
Supplement: Supplementary file 3 — Description of Additional Supplementary Materials [file 42003_2025_7971_MOESM3_ESM.pdf]

Description of Additional Supplementary Files

File name: Supplementary Data 1

Description: the source data behind all graphs in the paper (main article and supplementary material)
